# Involvement of *Methoprene‐tolerant* and *Krüppel homolog 1* in juvenile hormone‐mediated vitellogenesis of female *Liposcelis entomophila* (End.) (Psocoptera: Liposcelididae)

**DOI:** 10.1002/arch.21973

**Published:** 2022-10-03

**Authors:** Bin‐Bin Yang, Shi‐Yuan Miao, Yu‐Jie Lu, Sui‐Sui Wang, Zheng‐Yan Wang, Ya‐Ru Zhao

**Affiliations:** ^1^ School of Grain Science and Technology Jiangsu University of Science and Technology Zhenjiang China; ^2^ School of Food Science and Technology Henan University of Technology Zhengzhou China; ^3^ College of Science, Health, Engineering and Education Murdoch University Murdoch Western Australia Australia

**Keywords:** Jjuvenile hormone, *Krüppel homolog 1*, *methoprene‐tolerant*, psocid, reproduction, RNA interference

## Abstract

Methoprene‐tolerant (Met) as an intracellular receptor of juvenile hormone (JH) and the *Krüppel‐homolog 1* (*Kr‐h1*) as a JH‐inducible transcription factor had been proved to contribute to insect reproduction. Their functions vary in different insect orders, however, they are not clear in Psocoptera. In this study, *LeMet* and *LeKr‐h1* were identified and their roles in vitellogenesis and ovarian development were investigated in *Liposcelis entomophila* (Enderlein). Treatment with exogenous JH III significantly induced the expression of *LeKr‐h1, LeVg*, and *LeVgR*. Furthermore, silencing *LeMet* and *LeKr‐h1* remarkably reduced the transcription of *LeVg* and *LeVgR*, disrupted the production of Vg in fat body and the uptake of Vg by oocytes, and ultimately led to a decline in fecundity. The results indicated that the JH signaling pathway was essential to the reproductive process of this species. Interestingly, knockdown of *LeMet* or *LeKr‐h1* also resulted in fluctuations in the expression of *FoxO*, indicating the complex regulatory interactions between different hormone factors. Besides, knockdown of both *LeMet* and *LeKr‐h1* significantly increased *L. entomophila* mortality. Our study provides initial insight into the roles of JH signaling in the female reproduction of psocids and provided evidence that RNAi‐mediated knockdown of *Met* or *Kr‐h1* is a potential pest control strategy.

## Highlights

- *LeMet* and *LeKr‐h1* were identified in a Psocoptera insect.
- Juvenile hormone (JH) signaling pathway significantly affected vitellogenesis of female *Liposcelis entomophila*.
- Methoprene‐tolerant (Met) and *Krüppel‐homolog 1* (*Kr‐h1*) could act as potential targets for RNAi‐mediated psocid control.

## Graphical abstract

A graphical summary of this study and a model of the role of juvenile hormone (JH) signaling in the reproduction of *Liposcelis entomophila*. JH signaling induces reproduction by enhancing the expression of Vg through Met and Kr‐h1. Blocking JH signaling by knockdown with *Met* and *Kr‐h1*, which inhibits *Vg* expression and affects reproduction, can provide technical support for its control.

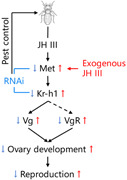

## 1 INTRODUCTION

Two main hormones, juvenile hormone (JH) and 20‐hydroxyecdysone (20E), regulate insect development, metamorphosis, and reproduction (Jindra et al., 2013; Roy et al., 2018; Wu, Yang, et al., 2020). The molecular basis of JH biosynthesis pathway and the prevention of 20E‐induced metamorphosis has been extensively studied (Belles, 2020; Song et al., 2014). The interaction of lower JH with higher 20E titers could induce molting behavior during the final instar larval or nymph growth stages (Kayukawa et al., 2017). In adults, JH is re‐elevated and fulfills its other major functions during reproduction, such as vitellogenesis and oogenesis (Raikhel et al., 2005). Since JH occurs even in wingless hemimetabolous insects, prompting reproduction is thought to be the evolutionarily older of the JH roles (Truman, 2019). Therefore, exploring the effects of JH on reproduction is important to understanding the physiology of insect reproduction.

The JH signaling pathway begins with JH being secreted by the corpus allatum (CA) and then binds with its receptor, methoprene‐tolerant (Met) (Jindra et al., 2013). Met subsequently recruits its partner Taiman (Tai) (also called SRC or FISC) to form the JH/Met/Tai complex, resulting in the activation of downstream signaling (Miyakawa et al., 2017; Roy et al., 2018; Santos et al., 2019). In hemimetabolous and holometabolous insects, Met conducts almost all physiological processes induced by JH, from the antimetamorphic action to the regulation of mating and sex pheromone production, Vg mRNA levels, and lipid metabolism, among others (Konopova et al., 2011; Lozano & Belles, 2011; Marchal et al., 2014; Minakuchi et al., 2008, 2009; Z. Zou et al., 2013). The realization of multiple functions of JH may be controlled through the different components of the Met cooperation (Marchal et al., 2014). In a previous study, *Met* mutation delayed ovarian development and decreased the fecundity of *Drosophila melanogaster* (Wilson & Fabian, 1986; Wilson et al., 2003). It indicates that *Met*‐mediated JH signaling may be involved in the regulation of insect reproduction. *Krüppel homolog 1* (*Kr‐h1*) plays a key downstream role in the JH pathway as a transductor of JH activity (Belles & Santos, 2014). The JH/Met/Tai complex induces *Kr‐h1* expression by binding directly to E‐box‐like motifs (Kayukawa et al., 2012). The role of *Kr‐h1* as an antimetamorphic factor has been extensively researched in many insects (Belles, 2020). Recently, researchers have turned their attention to the functions of *Kr‐h1* in female insect reproduction. Growing evidence from multiple species based on RNA interference (RNAi) suggests that *Met* and *Kr‐h1* are involved in vitellogenesis, including both basal hemimetabolous insects, such as Orthoptera (*Locusta migratoria* [Song et al., 2014] and *Schistocerca gregaria* [Gijbels et al., 2019]), Blattaria (*Blattella germanica* [Naghdi et al., 2016] and *Diploptera punctata* [Marchal et al., 2014]), Hemiptera (*Nilaparvata lugens* [Lin et al., 2015]), and most holometabolous insects, such as Diptera (*Aedes aegypti* [J. Zhu & Noriega, 2016] and *Bactrocera dorsalis* [Yue et al., 2018]), Coleoptera (*Tribolium castaneum* [Parthasarathy et al., 2010]) and Lepidoptera (*Helicoverpa armigera* [Ma et al., 2018; Zhang et al., 2018] and *Chilo suppressalis* [L. Miao et al., 2020; Tang et al., 2020]). However, the link between *Met* or *Kr‐h1* with vitellogenesis has not been studied in Psocoptera insects.

JH is the principal gonadotropic hormone controlling female reproduction in hemimetabolous insects (Khalid et al., 2021; Roy et al., 2018; Santos et al., 2019; Wu, Yang, et al., 2020). Besides, relatively relative more research progress has been made on the function of nutrition pathways (insulin‐like peptide [ILP] and target of rapamycin [TOR] signaling) on vitellogenesis in different species (Roy et al., 2018; Wu, Yang, et al., 2020). RNAi‐mediated silencing of *InR* (ILPs receptor) causes significant inhibition of JH biosynthesis, *Vg* expression, and ovarian development in female *B. germanica* (Abrisqueta et al., 2014). The ILP pathway can interact with JH receptor complex in addition to controlling the synthesis and secretion of JH. In *L. migratoria*, ILP pathway can help JH to stimulate fat body cells polyploidization, which accelerates massive Vg synthesis for synchronous maturation of multiple eggs (Guo et al., 2014; Wu et al., 2016; Wu, He, et al., 2020). Therefore, the molecular basis of JH and nutritional pathway crosstalk in regulating insect reproduction needs to be further explored.

The genus *Liposcelis* (Psocoptera: Liposcelididae) have become a major risk to global food security and safety (Ahmedani et al., 2010), especially *Liposcelis entomophila* (Enderlein) in tropical and subtropical regions (Nayak et al. 2014). The rampancy of resistant psocids may be due to the unreasonable application of insecticides (Bai et al., 2020; Nayak et al., 2014). A recent study suggests that sublethal treatment of phosphine will promote the fecundity of *L. entomophila* (Lu et al., 2020). This phenomenon has also been found in the chemical control of *N. lugens* (Wu, Ge, et al., 2020). Our previous study has shown that JH III significantly induced the expression of the *vitellogenin* (*Vg*) gene and affected the oviposition of *L. entomophila* (S. Y. Miao et al., 2021). Insect reproduction has been a focus of research in pest control, but relevant information is limited in *L. entomophila*. In this study, cDNAs encoding *LeMet* and *LeKr‐h1* were identified and the relative expression of *LeMet* and *LeKr‐h1* was determined at different stages throughout development using RT‐qPCR. The role of *LeMet* and its downstream target *LeKr‐h1* in the reproduction of *L. entomophila* was investigated using RNA interference and exogenous JHIII treatment. This study confirmed that these transcription factors are key factors in the JH signaling pathway and suggest the important role of JH in vitellogenesis and ovarian development of *L. entomophila*. It has laid the foundation for further exploration of the role of JH in the regulation of *L. entomophila* reproduction, which may help to effectively control the rampant of this pest.

## 2 MATERIALS AND METHODS

### 2.1 Insect rearing

The *L. entomophila* population used in this study was originally collected from a grain warehouse in Zhanjiang, Guangdong Province, China, in 2011. The colony was reared on an artificial diet consisting of wholewheat flour, skimmed milk, and yeast powder (10:10:1) (Leong & Ho, 1990) in a climatic chamber at 28 ± 1°C with 75 ± 5% relative humidity (RH) in continuous darkness.

### 2.2 RNA isolation and cDNA synthesis

Total RNA was extracted from the whole body or different tissues of female adults using *AxyPrep* Multisource Total RNA Miniprep Kit (Axygen) according to the manufacturer's protocol. To investigate the developmental expression profiles of *LeMet* and *LeKr‐h1*, individuals were collected from eggs and nymphs (1st, 2nd, 3rd, and 4th instars), females and males within 1 week after emergence, and adult females on different days after emergence. Tissues of adult females within 1 week after emergence (including head, thorax, midgut, ovary, and fat body) were dissected in phosphate‐buffered saline (PBS). All samples were frozen immediately in liquid nitrogen and stored at −80°C until RNA extraction. The extracted RNA was instantly dissolved in TE buffer and checked for quality, concentration, and purity at an absorbance ratio of optical density (OD) 260/280 (1.8–2.1) on a BioPhotometer (Eppendorf). Each experiment contained 30 individuals and was performed in at least three biological replicates. Finally, first‐strand cDNA was synthesized from 1 µg total RNA using a PrimeScript™ RT reagent kit with gDNA Eraser (Perfect Real Time) (Takara), following the manufacturer's protocol. The synthesized first‐strand cDNA was stored at –20°C until further processing.

### 2.3 Amplification of the full‐length mRNA of *LeMet* and *LeKr‐h1*

The sequence of *LeMet* and *LeKr‐h1* was obtained from transcriptome datasets and confirmed by checking its homology against other family members using the BLAST tools of the National Center for Biotechnology Information (NCBI) (https://blast.ncbi.nlm.nih.gov/Blast.cgi). Gene‐specific primers for amplification of gene full‐length coding regions were designed using the Primer‐BLAST tool (https://www.ncbi.nlm.nih.gov/tools/primerblast/) (Table 1). The PCR program was 95°C for 3 min, 35 cycles of denaturing at 98°C for 10 s, annealing at 56°C–62°C (based on the primers' annealing temperature) for 5 s and extension at 72°C for 45 s, with a final extension at 72°C for 2 min using PrimeSTAR Max DNA polymerase (Takara) according to manufacturer's protocol. The PCR amplification products were then electrophoresed on a 1% agarose gel, extracted, purified with a gel purification kit (Axygen), and cloned into a TA cloning vector pMD19‐T (Takara), and transformed into *Escherichia coli* DH5a competent cells (Sangon Biotech Co., Ltd) according to the instructions of the manufacturer. Positive clones were confirmed using PCR for sequencing (Sangon Biotech Co., Ltd). The nucleotide and deduced amino acid sequences were reconfirmed using the BLAST server of NCBI. Sequences were predicted by the ORF finder (http://www.ncbi.nlm.nih.gov/gorf/orfig.cgi). Tools from the ExPASy proteomics server (http://www.expasy. org) were used to deduce putative amino acid sequences from the corresponding sequences. The isoelectric point (pI) and molecular weight (Mw) of *LeMet* and *LeKr‐h1* were calculated using the Compute pI/Mw tool (http://web.expasy.org/protparam/). The modular domains of *LeMet* and *LeKr‐h1* were analyzed with the SMART program (http://smart.embl-heidelberg.de/).

**Table 1:** Primers used in this study for cloning, RT‐qPCR, and dsRNA synthesis in *Liposcelis entomophila*

Purpose	Primer name	Sequence (5′–3′)	
Coding sequence amplification	*Met‐F*		TCGCGGACGAAATGAGTTGA
	*Met‐R*		GCACGTTAAATCAGACTTCATGGT
	*Krh1‐F*		ATGACTGGAGTGAAAGAAGAAGTTC
	*Krh1‐R*		TTAGGTCTTTTGTTGCAGGGTAGG
dsRNA synthesis	*T7‐GFP‐F*		TAATACGACTCACTATAGGGAGGACGACGGCAACTACAAG
	*T7‐GFP‐R*		TAATACGACTCACTATAGGGCTTGTCGACGGAGCTCGAAT
	*T7‐Met‐F*		TAATACGACTCACTATAGGGGAAAGAACGCCAGTTCGTCG
	*T7‐Met‐R*		TAATACGACTCACTATAGGGGGCCCATTAGCTCGTTCTGT
	*T7‐Krh1‐F*		TAATACGACTCACTATAGGGCGGGAAGTTACACAGGCACA
	*T7‐Krh1‐R*		TAATACGACTCACTATAGGGCACGTCCGAATTTTGCGCTT
Quantitative real‐time PCR (RT‐qPCR)	*qLeMet‐F*		ACGTCTTCCTCGGCTCAAAG
	*qLeMet‐R*		TAAAAGGATCTCCGCTGCCG
	*qLeKrh1‐F*		TCAAGCGGAAGCAGCCATTA
	*qLekrh1‐R*		CACCGTCCTTGCTGTGAGAT
	*qLeVg‐F*		GAAGAATGGGTACAACAAAC
	*qLeVg‐R*		GTTCGGCGATCTTTTCAGCG
	*qLeVgR‐F*		CGCATCCGTTTTCGATTGCT
	*qLeVgR‐R*		ACTCGTGGTTGTAAGGCTGG
	*β‐actin‐F*		CTTGACGGAGCGTGGTTATT
	*β‐actin‐R*		ACCGATGGTGATTACTTGACC
	*qLeJHAMT‐F*		TCGCTCAGTTTGACATCGCC
	*qLeJHAMT‐R*		AGGCTCACCACCGCTAATCT
	*qLeInR‐F*		AGTCGTGTCGGAAGGTCAAC
	*qLeInR‐R*		TCCGTCGGCTATTTCGATGG
	*qLeFoxO‐F*		AGACTTTCCCGGGTGGTTTC
	*qLeFoxO‐R*		TGACTGTTTGCCCTCCTGAC

### 2.4 Sequence comparisons and phylogenetic analysis

The BLASTx algorithm was employed to run the similarity searches. The amino acid sequences of *LeMet* and *LeKr‐h1* were aligned with those of other insects using the DNAMAN 6.0 software package (Lynnon Corporation), and the putative complete coding sequences were submitted to GenBank.

Phylogenetic relationships between LeMet or LeKr‐h1 and other insects were investigated by first aligning the respective amino acid sequences with *ClustalW* software, then using MEGA X software to construct two neighbor‐joining phylogenetic trees with a *p*‐distance model and pairwise deletion of gaps, and each presented with a total of 1000 bootstrap replications were run to test topology (Kumar et al., 2018).

### 2.5 Expression profiling analysis of *LeMet* and *LeKr‐h1*

Quantitative real‐time PCR (RT‐qPCR) was used to measure the tissue‐ and stage‐specific expression profile of *LeMet* and *LeKr‐h1*. The β‐actin gene of *L. entomophila* (GenBank accession no. MT603494.1) was used as a reference gene to normalize the target gene expression and to correct for sample‐to‐sample variation Specific qPCR primers were designed using the NCBI profile blast (http://www.ncbi.nlm.nih.gov/tools/primer-blast) for the experiment (Table 1) (S. Y. Miao et al., 2021). The reaction was performed in a 10 μl volume containing 0.4 μl of cDNA, 5 μl of 2 × TB Green™ Premix Ex Taq II (Tli RNaseH Plus) (Takara), 0.3 μl of each primer (10 mM), and 4 μl of RNase‐free H_2_O and tested on a LightCycler 96 instrument (Roche Diagnostics, Basel) using the following protocol: 95°C for 2 min (for predenaturation), 40 cycles of 95°C for 15 s, and 60°C for 30 s (for fragment amplification and signal collection, respectively), and 60°C–95°C with 0.5°C increasing per 5 s (for dissociation and melting curve analysis on the specific amplification). Each sample had at least there technical replicates. The relative expression levels of *LeMet, LeKr‐h1*, or *LeVg* were normalized by the mRNA abundance of β‐actin using the 2^−ΔΔCT^ method (Schmittgen & Livak, 2008).

### 2.6 RNA interference and JH III treatment

RNAi was used to investigate the function of JH signaling pathway gene in *L. entomophila* ovarian development by knocking down *LeMet* and *LeKr‐h1*. The primers for the RNAi experiment were listed in Table 1 and designed to amplify *LeMet* and *LeKr‐h1* fragments including a T7 promoter region in the sense and antisense strands. The *green fluorescent protein* gene (*GFP*) was used from the lab‐owned as a heterologous control (Wang et al., 2021). The PCR‐amplified products were purified using a gel extraction kit (Omega bio‐tek) as templates for dsRNA synthesis. The corresponding dsRNA was synthesized in vitro using the TranscriptAid™ T7 High Yield Transcription Kit (Thermo Fischer Scientific). The integrity of dsRNA was measured using agarose gel electrophoresis, and the concentration was determined using a BioPhotometer. The final dsRNA products were stored at −80°C and used within 1 week.

The RNAi bioassay comprised a treatment group (*dsLeMet*‐fed or *dsLeKrh1*‐fed) and a control group (*dsGFP*‐fed). RNAi was performed as described (S. Y. Miao et al., 2021; Wei et al., 2020) and was further optimized as follows: (1) For each group, dsRNA dissolved in 200 μl of nuclease‐free water was added to a glass bottle (25 ml) containing 10 mg of sieved artificial diet. The final concentration of dsRNAs was 200 μg per mg of artificial diet. (2) The mixture was frozen into a solid state for 1 h in a −80°C refrigerator. The solid mixture in the glass bottles was then vacuumed and freeze‐dried (Labconco FreeZone 6 Plus) for 24h and balanced moisture to 75% RH. (3) In each treatment group, 50 newly emerged females were starved for 24 h, and an equal number of males were transferred to each glass bottle. (4) Feeding bioassays were performed at 28 ± 1°C, 75 ± 5% RH in complete darkness in an incubator. After 2 days of continuous feeding, the silencing efficiencies of the target genes were tested using RT‐qPCR.

To examine the effect of *LeMet* and *LeKr‐h1* RNAi on survivability and oviposition was detected by calculating the survival rate, total number of eggs produced, and hatching rate by each group within 5 days after continuous feeding of dsRNA. In addition, in each treatment, the ovarian phenotypes of females were observed. The images of dissected ovaries of the remaining live psocids from the gene silencing experiment were taken under an Olympus CKX41 Microscope equipped with a DP27 digital camera (Olympus). The images were processed with Adobe Photoshop CC2019 (Adobe Systems Software Ireland Ltd).

JH treatments were performed to assess whether the expression of *Lemet* and *Lekr‐h1* were JH‐dependent. A dose of 10 µg of JH III (Toronto Research Chemicals) was diluted in analytical grade acetone at a concentration of 10 µg/µl. A volume of 23 nl of JH III solution was topically applied on the abdominal tergites of 7‐day‐old virgin female adults using a Nanoinjector (Drummond Scientific). Controls were equivalently treated with acetone. Dissections for mRNA measurements were carried out 4, 6, and 8 h later, respectively. Each of the above samples had three replicates.

### 2.7 Statistical analysis

Statistical analyses of differences in mRNA expression and fecundity were performed using GraphPad Prism 8 software package. One‐way analysis of variance followed by Tukey's HSD multiple comparison test was applied to test for significant differences among different developmental stages or tissues. An independent samples *t*‐test (*p* < 0.05 and *p* < 0.01) was used to determine the significance of differences between the treatment and control in the dsRNA feeding assay. All data are expressed as mean ± standard error (SE).

## 3 RESULTS

### 3.1 Sequence analysis of *LeMet* and *LeKr‐h1*

The coding sequence (CDS) of *LeMet* is 2,493 bp long and encodes an 830 amino acid protein with a calculated molecular weight (MW) of 92.89 kDa and a theoretical isoelectric point (pI) of 5.99. *LeKr‐h1* has a 1647 bp CDS encoding a 548 amino acid protein with a calculated MW of 61.96 kDa and a theoretical pI of 8.41. The nucleotide sequences and the predicted amino acid sequences were deposited in NCBI Genbank (accession number: MZ054395 and OK274053) (Figure 1a).

Figure 1Identification and sequence analysis of *Liposcelis entomophila* methoprene‐tolerant (LeMet) and Krüppel‐homolog 1 (LeKr‐h1). (a) Diagram of deduced domains of LeMet and LeKr‐h1 predicted by the software package illustrator of biological sequences version 1.0 (http://ibs.biocuckoo.org/). (b) Alignment of amino acid sequences of LeMet with those of other insects; DpMet (*Diploptera punctata*, AIM47235.1), BgMet (*Blattella germanica*, CDO33887.1), and ZnMet (*Zootermopsis nevadensis*, BAR92640.1). Specific domains of Met family are indicated and marked with black lines above the sequence. (c) Alignment of amino acid sequences of LeKr‐h1 with those of other insects; NLKr‐h1 (*Nilaparvata lugens*, AHC57980.1), LmKr‐h1 (*Locusta migratoria*, AHX81747.1), and SfKr‐h1 (*Sogatella furcifera*, QHD5540.1). Specific domains are indicated and marked with black lines above the sequence.

**Figure:**
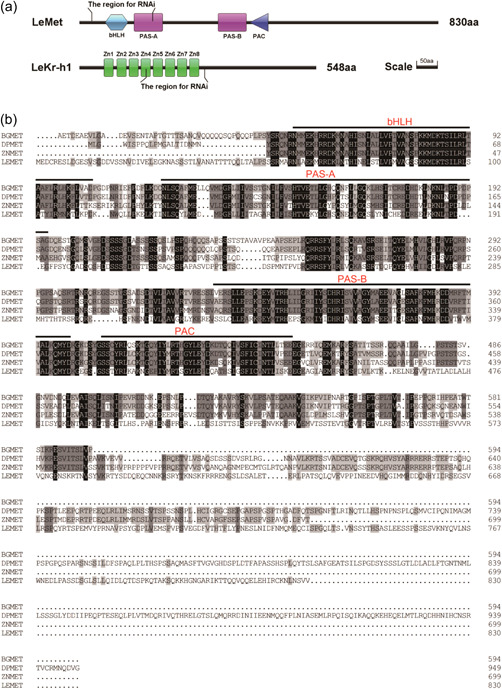

**Figure:**
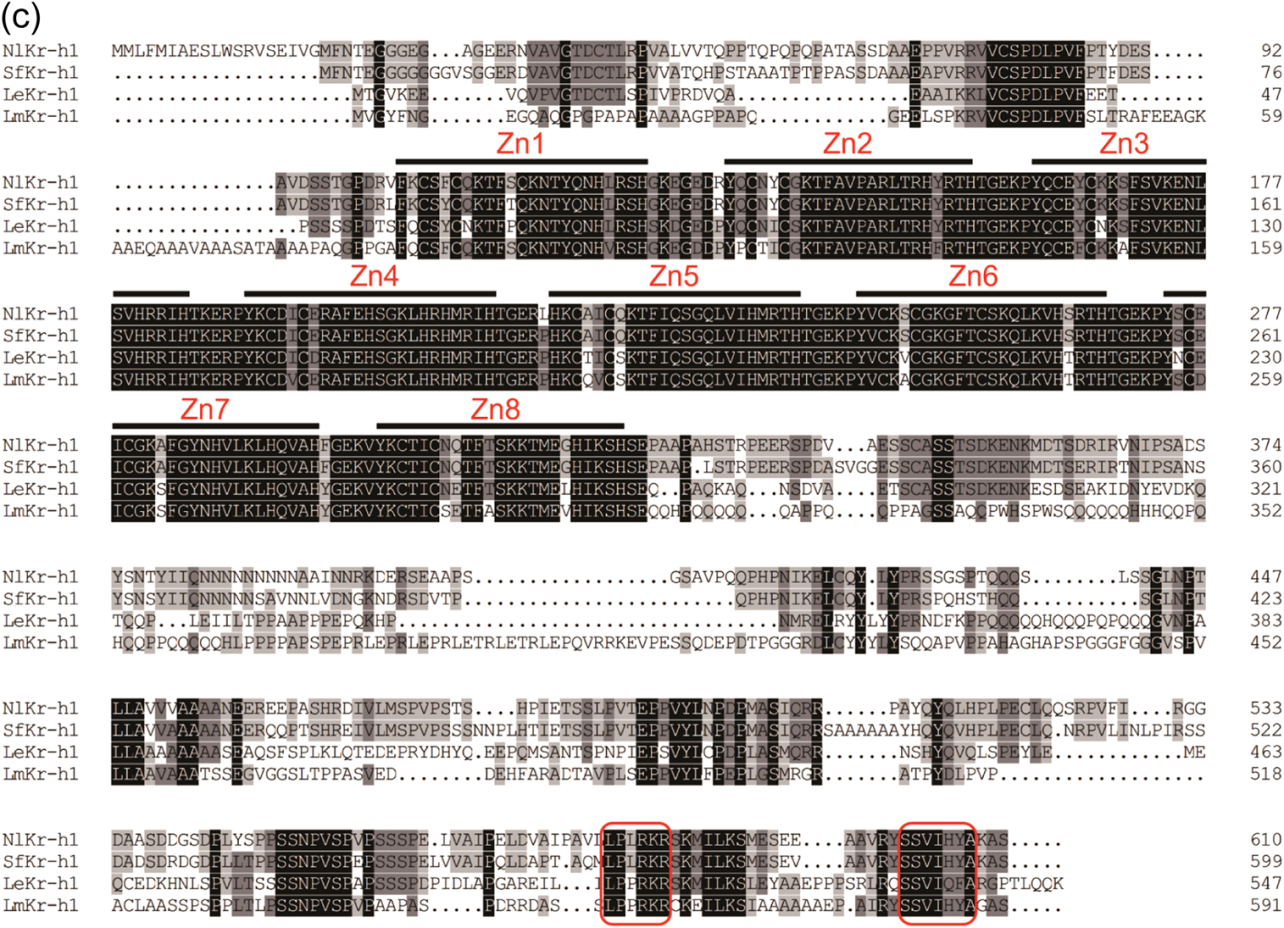

The deduced amino acid sequence of *LeMet* contains three typical functional domains of bHLH‐PAS protein family: bHLH (a.a. 60–113), PAS‐A (a.a. 127–193), and PAS‐B (a.a. 321–387) (Figure 1b). A PAS‐associated C‐terminal (PAC) region was found at the C‐terminal of PAS‐B domain. LeKr‐h1 has a DNA binding motif composed of eight putative C_2_H_2_‐type zinc fingers, a typical structure feature of *Kr‐h1* protein. The first of domains (Zn1) is less conserved than the other seven (Zn2–Zn8) which are highly conserved (Figure 1c).

**Figure 2:**
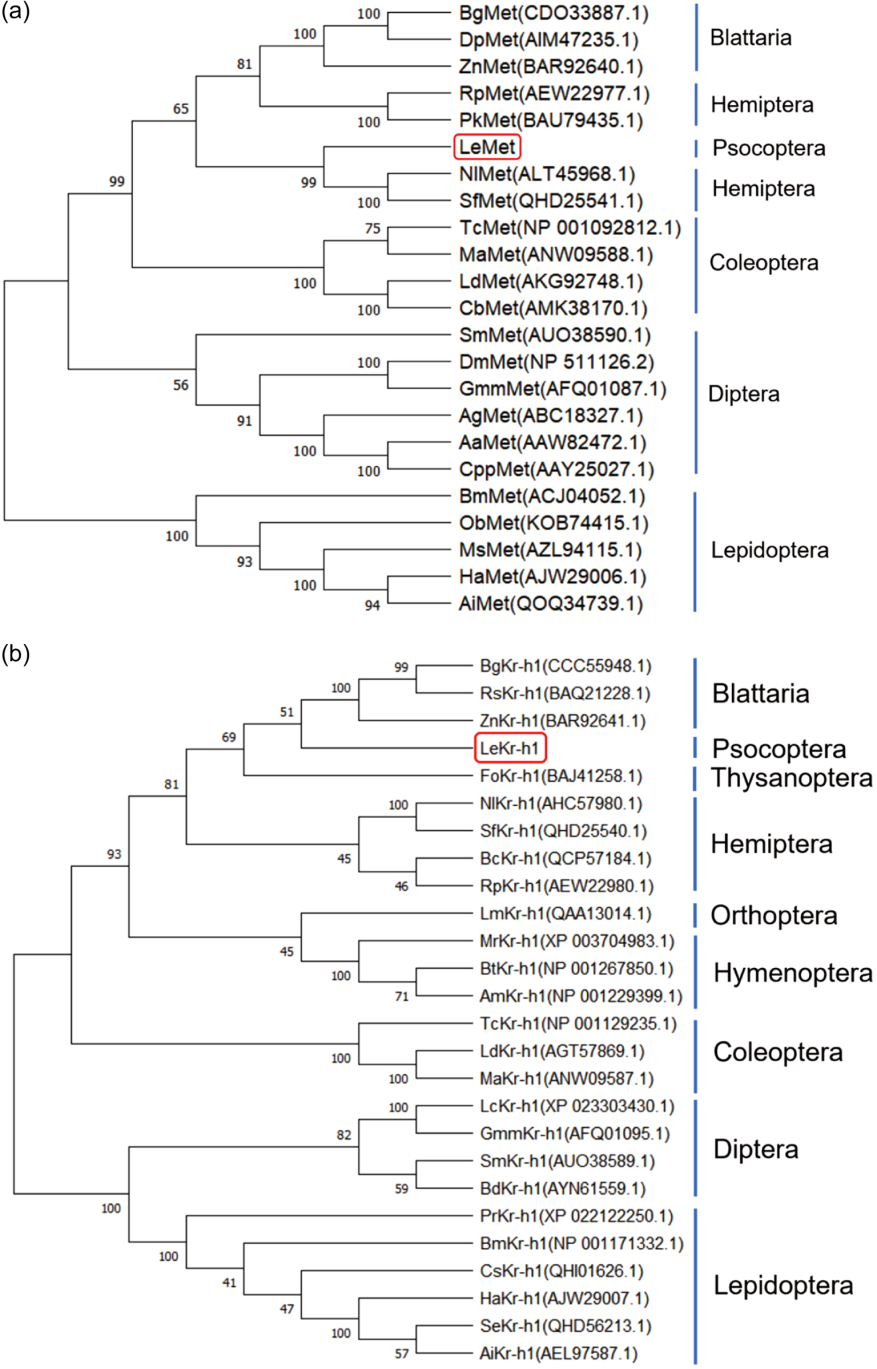
The neighbor‐joining phylogenetic tree analysis was built by MEGA X software for methoprene‐tolerant (Met) (a) and Krüppel‐homolog 1 (Kr‐h1) (b) from *Liposcelis entomophila* and other insects. Numbers at the nodes of branches indicate bootstrap values (%) based on 1000 replicates. Numbers following each protein name are the GenBank accession numbers for each species' protein. The red wireframe represents the protein of *L. entomophila*. BgMet and BgKr‐h1, *Blattella germanica*; DpMet, *Diploptera punctata*; ZnMet and ZnKr‐h1, *Zootermopsis nevadensis*; RsKr‐h1, *Reticulitermes speratus*; RpMet and RpKr‐h1, *Rhodnius prolixus*; PkMet, *Planococcus kraunhiae*; NlMet and NlKr‐h1, *Nilaparvata lugens*; BcKr‐h1, *Bactericera cockerelli*; SfMet and SfKr‐h1, *Sogatella furcifera*; TcMet and TcKr‐h1, *Tribolium castaneum*; MaMet and MaKr‐h1, *Monochamus alternatus*; LdMet and LdKr‐h1, *Leptinotarsa decemlineata*; CbMet, *Colaphellus bowringi*; FoKr‐h1, *Frankliniella occidentalis*; LmKr‐h1, *Locusta migratoria*; MrKr‐h1, *Megachile rotundata*; BtKr‐h1, *Bombus terrestris*; AmKr‐h1, *Apis mellifera*; SmMet and SmKr‐h1, *Sitodiplosis mosellana*; GmmMet and GmmKr‐h1, *Glossina morsitans morsitans*; DmMet, *Drosophila melanogaster*; AgMet, *Anopheles gambiae*; AaMet, *Aedes aegypti*; LcKr‐h1, *Lucilia cuprina*; CppMet, *Culex pipiens pipiens*; BdKr‐h1, *Bactrocera dorsalis*; BmMet and BmKr‐h1, *Bombyx mori*; ObMet, *Operophtera brumata*; MsMet, *Mythimna separata*; PrKr‐h1, *Pieris rapae*; HaMet and HaKr‐h1, *Helicoverpa armigera*; AiMet and AiKr‐h1, *Agrotis ipsilon*; CsKr‐h1, *Chilo suppressalis*; SeKr‐h1, *Spodoptera exigua*.

Based on the predicted protein sequences of Met and Kr‐h1 homologs from various insect species. Phylogenetic analysis indicated that Mets and Kr‐h1s from hemimetabolous were separately grouped (Figure 2). The results were consistent with the classical taxonomic divergence. *LeMet* has the highest amino acid identity with *D. punctata* Met (48.10%), and *LeKr‐h1* shares high similarity to the *Kr‐h1s* of other Blattaria; the highest sequence similarity was with *L. migratoria* Kr‐h1(76.81%)(Figure 1b,c). These results suggest that Met and Kr‐h1 sequences are evolutionarily more closely related to hemimetabolous insects than to other insects.

### 3.2 Expression profiles of *LeMet* and *LeKr‐h1* in the develpmental stages and tissues

During the early developmental stages of *L. entomophila, LeMet, LeKr‐h1, and LeVg* were all highly expressed in the embryonic stage (egg). It shows that *LeMet* and *LeKr‐h1* may play roles in the embryonic development of *L. entomophila*. The expression of *LeKr‐h1* and *LeVg* were decreased with increasing age (from first‐ to fourth‐instar nymphs) and the expression of *LeVg* was picked up in the final instars. This may be the result of *Kr‐h1*‐mediated antimetamorphic action of JH. mRNA levels of Le*Met* did not show significant changes throughout the nymph stage (Figure 3d). There were no significant differences in the expression of *LeMet* and *LeKr‐h1* in males and females within 1 week after emergence. The expression of female *LeMet* was fluctuations after emergence (Figure 3b). The expression pattern of *LeKr‐h1* and *LeVg* gradually increased and reached the first peak at 12 days (Figure 3c), hinting that Kr‐h1 is necessary for vitellogenesis in *L. entomophila*. Nevertheless, *LeVg* had a notably higher expression in female adults than in male adults (Figure 3a), suggesting that the JH signaling pathway plays certain physiological functions in male adults. Expression of *LeMet, LeKr‐h1, and LeVg* could be detected in the main tissues of female adults of *L. entomophila* with similar expression patterns, with the highest expression in the fat body, followed by the head and thorax, and with the lowest expression in the ovary and midgut (Figure 3e). This suggests that JH stimulates the synthesis of *Vg* primarily in the fat body.

Figure 3Spatio‐temporal expression of *LeMet, LeKr‐h1*, and *LeVg*. (a) Relative expression levels of LeMet and LeKr‐h1 in male and female adults. (b) Relative expression levels of *LeMet* at different times after emergence. (c) Relative expression levels of *LeKr‐h1* and *LeVg* in females at different times after emergence. The correlation coefficient (*r*) between the measurements was found via a Pearson correlation calculation. (d) Relative expression levels of *LeMet, LeKr‐h1* and *LeVg* in egg stage and 1st to 4th instar nymphal instars (N1, N2, N3, and N4). (e) Relative expression levels of LeMet in various tissues of females. head, thorax, fat body, and midgut. Bars indicate the mean (±SE) of three biological replicates. Different letters above bars represent significant differences (analysis of variance followed by Tukey's test, *p* < 0.05).

**Figure:**
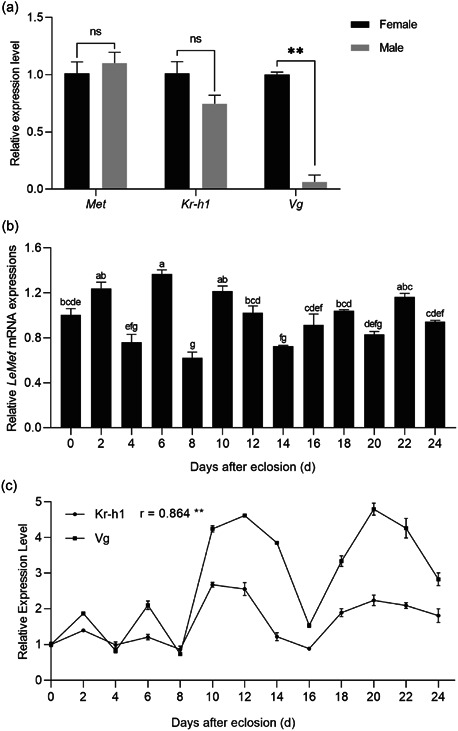

**Figure:**
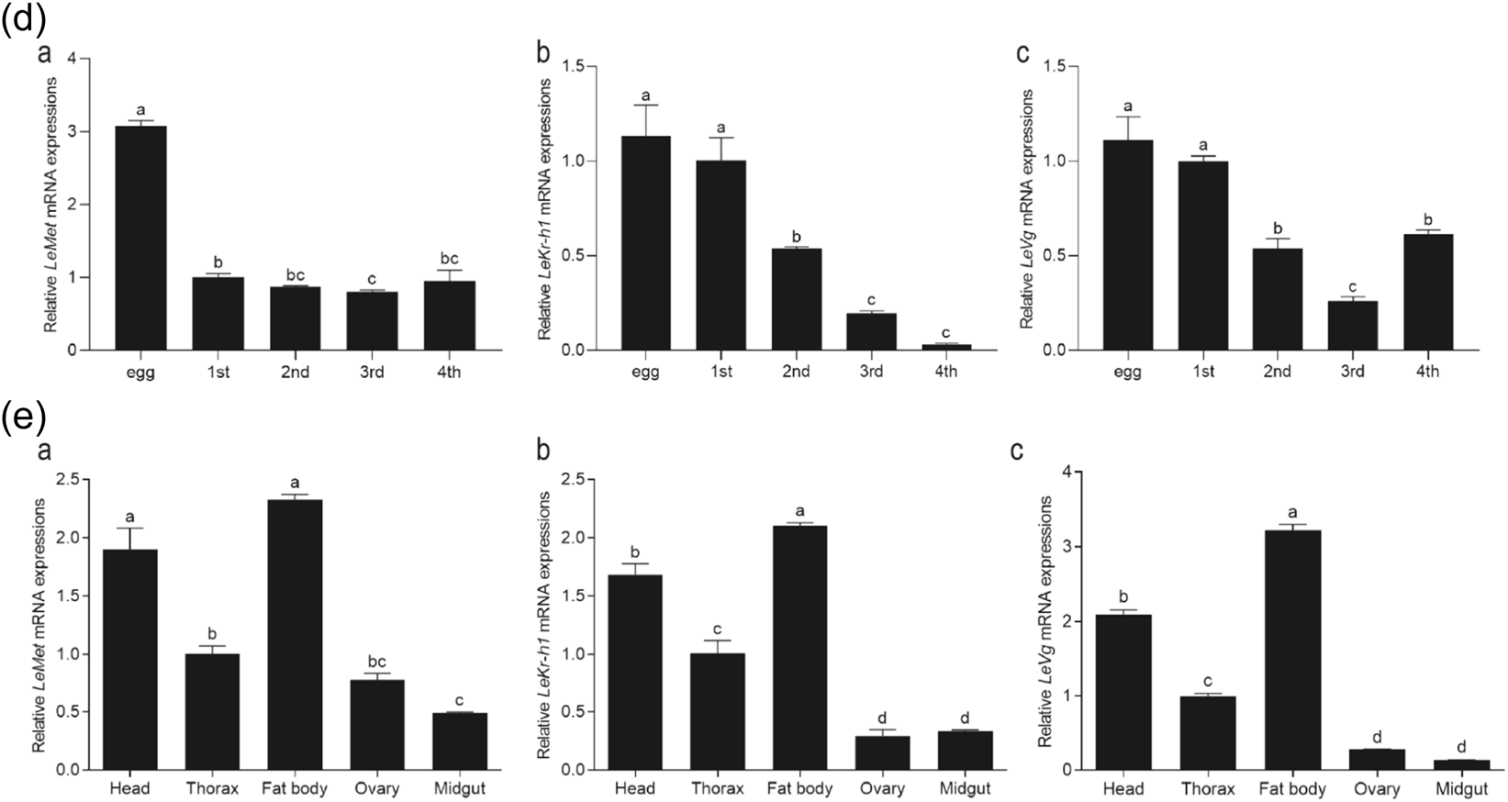

### 3.3 Exogenous JH III treatment on the expression of *LeMet* and *LeKr‐h1*

**Figure 4:**
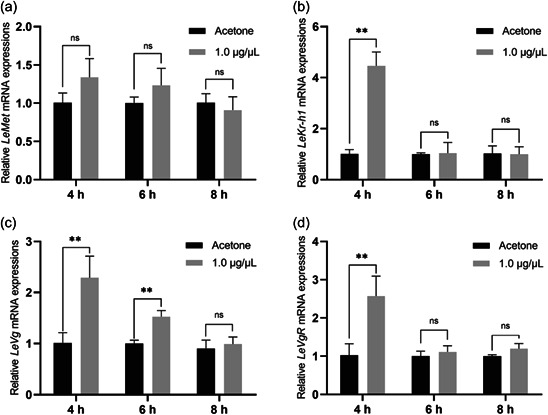
Expression profiles of *LeMet* (aA), *LeKr‐h1* (b), *LeVg* (c), and *LeVgR* (d) at 4, 6, and 8 h time points after application of juvenile hormone (JH) III of female adults within 2 days of emergence. The expression level of each treatment was relative to that of acetone control, which was arbitrarily set at 1. Bars represent the means ± SE. Values followed by different letters within each group were significantly different by Tukey's HSD test (**p < 0.05).

To examine whether *LeMet* and *LeKr‐h1* were regulated by JH, exogenous JH III was applied to the abdomen of *L. entomophila*. Overall, the induction effect of JH was progressively reduced with increasing time. *LeMet* expression was elevated but not significantly different at 4 and 6 h of treatment groups compared to that in the control group. However, *LeKr‐h1* expression was 4.42‐fold higher after 4 h of treatment (Figure 4). Meanwhile, *LeVg* and *LeVgR* expression increased by 2.26 and 2.50 times at 4 h after treatment. These results indicate that *LeKr‐h1* is involved in the regulation of the expression of Vg in *L. entomophila*.

### 3.4 Functional analysis of *LeMet* and *LeKr‐h1* by RNA interference

**Figure 5:**
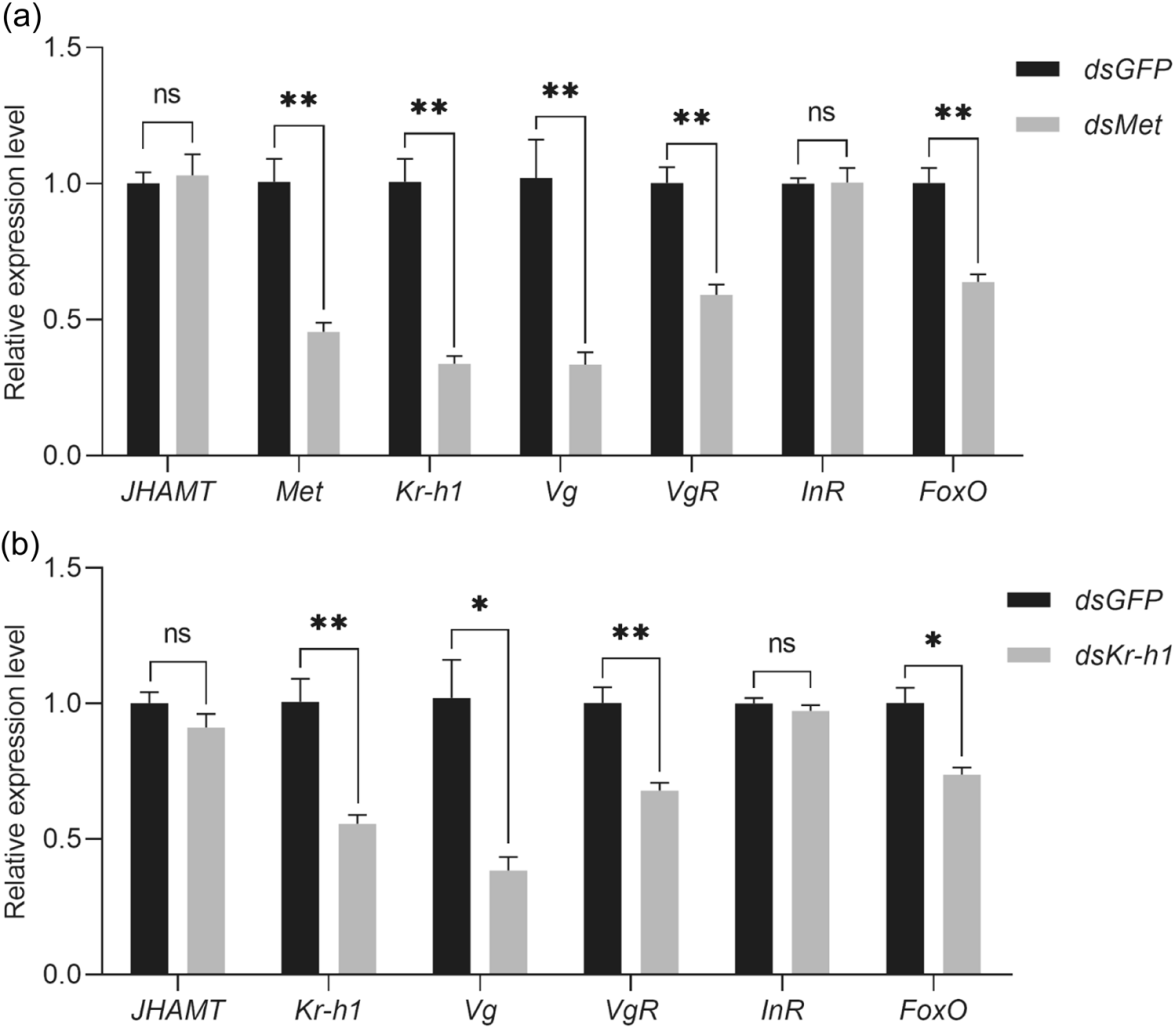
Relative expression of juvenile hormone (JH) pathway, 20E pathway, and insulin genes in 2 days after *LeMet* (a) and *LeKr‐h1* (b) knockdown. Error bars indicate SE; *p < 0.05; **p < 0.01 (*t*‐test). dsGFP, double‐stranded RNA for green fluorescent protein; FoxO, Forkhead box protein O; InR, insulin receptor; JHAMT, Juvenile hormone acid methyl transferase; Vg, vitellogenin; VgR, vitellogenin receptor.

**Figure 6:**
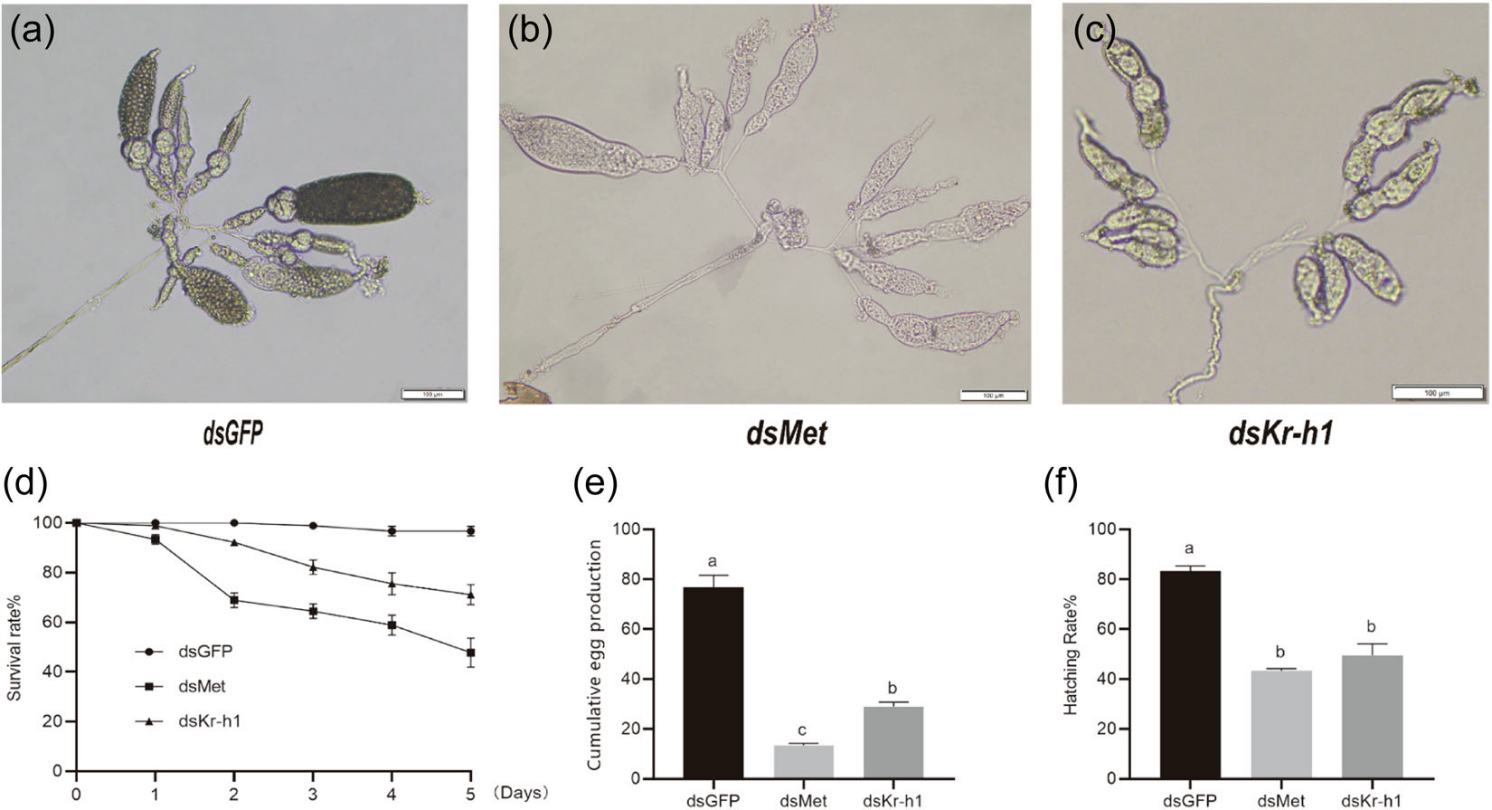
The evaluation of ovarian development and fertility after *LeMet* and *Krüppel‐homolog 1* (*Kr‐h1*) knockdown. (a–c) Ovaries were dissected and photographed under dissection microscopy 5 days after feeding. Double‐stranded RNA for green fluorescent protein (dsGFP) was injected as a negative control. (d) Mortality of RNA interference within 5 days. (e) Cumulative spawning after 5 days of RNA interference. (f) Hatching rate of eggs after different dsRNA treatments.

The *LeMet* and *LeKr‐h1* transcriptions were significantly inhibited after 2 days of feeding dsRNA‐containing diet compared with the control groups (*dsGFP*) (54.45% and 44.36% less, respectively) (Figure 5). Meanwhile, *LeVg* and *LeVgR* transcription were significantly reduced (Figure 5). Yolk protein deposition was significantly reduced in both *dsMet* and *dsKr‐h1*‐treated females, whereas ovarian development was normal in *dsGFP*‐treated females (Figure 6). Survival of female adults gradually decreased after gene disturbance by dsMet or dsKr‐h1 (Figure 6d). In addition, the cumulative egg production and hatching rates were significantly decreased in the *dsMet*‐ and *dsKr‐h1*‐fed groups compared with the *dsGFP*‐fed group (Figure 6e,f). These data suggest that inhibition of *Kr‐h1* suppresses JH‐induced expression of *Vg* in the fat body of *L. entomophila* and blocks yolk deposition and ovarian development. These results demonstrate the importance of *LeMet* and *LeKr‐h1* in the female reproduction of *L. entomophila*.

Both knockdowns of *LeMet* and *LeKr‐h1* did not affect the expression of *LeJHAMT*, suggesting that the rate of JH synthesis may not have been affected (Figure 5). Insulin receptor (InR) and Forkhead box protein O (FoxO) are both involved in the ILPs signaling pathway. With *LeMet* and *LeKr‐h1* knockdown, both *LeInR* was almost unchanged in this pathway but the level of *FoxO* dropped by 36.36% and 26.55%, respectively (Figure 5). These results suggested that Kr‐h1 influenced the JH and ILPs signaling pathways.

## 4 DISCUSSION

The identification of Met and Kr‐h1 has been reported in different insect orders (Wu, Yang, et al., 2020), this study is the first report on these two genes from Psocoptera insects. The sequence alignment and conserved domain analysis showed that *LeMe*t contains three typical bHLH, PAS, and PAC domains and it is a homolog of JH intracellular receptors. The bHLH domain binds target DNA sites and plays an important role in the transcriptional induction of Kr‐h1 by JH III (Cui et al., 2014); The PAS domain contributes to forming JH receptor complexes and recognizing JHRE (Charles et al., 2011; Kayukawa & Shinoda, 2015; Li et al., 2011); The PAC domain may facilitate the dynamic interactions between Met and its coactivators (Moglich et al., 2009; Partch & Gardner, 2010). Multiple alignments revealed that *LeKr‐h1* contains eight putative C_2_H_2_‐type zinc‐finger domains (Zn1‐Zn8). Among that, the Zn1 domain was less conserved than the others, which was also found in *S. furcifera* (Hu et al., 2020), and *B. dorsalis* (Yue et al., 2018). Some crustaceans, such as *Daphnia pulex, Hyalella azteca*, and *Portunus trituberculatus*, have lost the Zn1 motif in their Kr‐h1s (Xie et al., 2018). These suggest that Zn1 may have little effect on the DNA binding capacity of Kr‐h1. The C‐terminal sequence of LeKr‐h1 contains two putative protein interaction motifs LPPRKR (a.a. 506–511) and SVIQFA (a.a. 534–539) (Figure 1c). It is shown that the unique structures of LeMet and LeKr‐h1 are essential in achieving the function of JH signaling transduction.

In this study, *LeMet* and *LeKr‐h1* were expressed at all developmental stages of *L. entomophila*, which is consistent with most insects (Cheng et al., 2020; Han et al., 2022; Lin et al., 2015; Yue et al., 2018). The expression of *LeMet* was significantly higher at the embryonic stage than that in the nymphal stages of *L. entomophila*, which indicated that *LeMet* expression may be associated with embryonic development in *L. entomophila*. JH is involved in the regulation of insect development, including mating, reproduction, aging, and polymorphism in insects (Marchal et al., 2014). However, the role of JH in insect embryos development is mysterious (Fernandez‐Nicolas & Belles, 2017). A recent study reported that Met was necessary for embryonic development in *T. castaneum* (Naruse et al., 2020). The involvement of *Met* in insect embryonic development remains to be investigated. *Kr‐h1* is the main effector of antideformation action of JH in holometabolous and hemimetabolous insects (Belles, 2020). The expression of *LeKr‐h1* decreased rapidly at the third and fourth nymphal instars indicating the antimetamorphic role of *Kr‐h1* in *L. entomophila*. This effect was also demonstrated in *B. germanica* (Lozano & Belles, 2011), *P. apterus* and *Rhodnius prolixus* (Konopova et al., 2011), where disturbance of Kr‐h1 in the late nymphal stage triggers precocious metamorphosis (Belles, 2020). The temporal profile of *LeKr‐h1* transcripts after emergence was dynamic, with *LeVg* expression dependent on *LeKr‐h1*, suggesting that *Kr‐h1* is an early‐inducible gene of *Vg*. However, *Met* mRNA is relatively stable with few major fluctuations. Similar results have been reported in *D. punctata, B. germanica*, and *S. gregaria* (Gijbels et al., 2019; Lozano & Belles, 2014; Marchal et al., 2014). Besides, the high expression of *Met, Kr‐h1*, and *Vg* in the fat body of adult females implies that the fat body is involved in the role of JH in regulating vitellogenesis (Roy et al., 2018; Wu, Yang, et al., 2020). Interestingly, *LeMet* and *LeKr‐h1* also had high expression levels in the head. A recent study showed that Vg was also detected in the thoraxes and heads of *Plutella xylostella* (M. M. Zou et al., 2020). It is speculated that the fat body cells of *L. entomophila* may be partially distributed in the thoraxes and heads (Hwangbo et al., 2004). JHs are synthesized and secreted by CA (Kotaki et al., 2009). Since the whole heads were sampled, rather than brain tissue exclusively, some contamination by the CA cannot be ruled out. Although *Met* and *Kr‐h1* of the JH signaling pathway are involved in vitellogenesis in insects, their expression in adult males also indicates their involvement in other physiological processes. In *Agrotis ipsilon* adult males, *Met* and *Kr‐h1* were involved in the regulation of JH signaling in the maturation of the male accessory glands (Gassias et al., 2021), and the modulation of pheromone processing and sexual behavior (Duportets et al., 2012). These results indicate that *Met* and *Kr‐h1* are key response genes in the JH signaling pathway, and their expression at appropriate timing is essential for their correct function.

Exogenous hormone treatments are common methods to explore the physiological and biochemical effects of hormones on insects (Pener & Lazarovici, 1979). Exogenous JH or its analogs significantly stimulated the expression of *Kr‐h1* in *B. germanica, T. castaneum, Bombyx mori*, and *A. aegypti* (Cui et al., 2014; Kayukawa et al., 2014; Konopova et al., 2011; Naghdi et al., 2016; Zhang et al., 2018). In *N. lugens* and *B. dorsalis*, exogenous JHA application could greatly affect ovarian development, suggesting that JH played an important role in the female reproduction of these insects (Lin et al., 2015; Yue et al., 2018). JH III treatment increased the transcription of *LeKr‐h1, LeVg*, and *LeVgR* within 4–6 h. This result confirmed that *LeKr‐h1* was an important transcription factor in vitellogenesis and female reproduction of *L. entomophila*. It is worth noting that Met was not dependent on the increase of JH, and the same phenomenon was shown in *B. germanica* (Naghdi et al., 2016). This is possible because of that *Met*, as a receptor of JH, is located upstream of *Kr‐h1* and may have been increased before the time of our observations.

The knockdown of *LeKr‐h1* or *LeMet* significantly inhibited the expression of *LeVg*, which is consistent with studies in *S. gregaria, N. lugens*, and *T. castaneum* (Gijbels et al., 2019; Lin et al., 2015; Parthasarathy et al., 2010). It also inhibited the uptake of yolk protein and significantly reduced the egg production and hatching rate of *L. entomophila*. These results indicated that *LeMet* and *LeKr‐h1* in the JH signaling pathway were involved in the promotion of ovarian development and the egg‐laying process. The knockdown of Mets reduced the transcription of *Kr‐h1s* and *Vgs*, decreased yolk accumulation, and inhibited ovarian development in *B. germanica, D. punctata*, and *Colaphellus bowringi* (Liu et al., 2016; Marchal et al., 2014; Naghdi et al., 2016). In *P. apterus* and *C. lectularius*, Knockdown of *Mets*, rather than *Kr‐h1s*, significantly reduced Vgs expression (Gujar & Palli, 2016; Smykal et al., 2014). These were probably due to incomplete silencing of *Kr‐h1s*. Moreover, knockdown of *Kr‐h1* would seriously decrease the egg hatching in *C. lectularius* (Gujar & Palli, 2016), indicating the other role of *Kr‐h1* in embryonic development. The reproduction regulation in some Lepidopteran, Hymenoptera, and Diptera insects is more complex than in hemimetabolous insects (Khalid et al., 2021; Roy et al., 2018). This indicates that the regulation of reproduction in different insects varies from species. Anyhow, current evidence suggests that JH regulates *LeKr‐h1* through receptor *LeMet*‐mediated signaling, which in turn regulates vitellogenesis and thus its fecundity in *L. entomophila*.

Nutrition‐related pathways have important roles in controlling the biosynthesis and secretion of JHs in insects, thereby influencing their reproductive events (Roy et al., 2018). Although *LeMet* and *LeKr‐h1* in JH signaling are critical for vitellogenesis in *L. entomophila*, it seems to have interaction with other signaling pathways. *FoxO* plays a critical role in mediating the crosstalk between insulin and JH signaling pathways to coordinate insect vitellogenesis (Koyama et al., 2013; Roy et al., 2018; Santos et al., 2019; Smykal & Raikhel, 2015). In *B. germanica, T. castaneum, A. aegypti, L. migratoria*, and *Maruca testulalis*, knockdown of *FoxO* significantly reduced *Vg* transcript levels and impaired oocyte maturation (Abrisqueta et al., 2014; Al Baki et al., 2019; Hansen et al., 2007; Parthasarathy & Palli, 2011; Wu, He, et al., 2020). In *T. castaneum*, JH signaling pathway could increase *ILP2* expression level via *Met*, subsequently leading to FoxO phosphorylation and ultimately depletion from the nucleus, which allows the expression of *Vg* (Sheng et al., 2011). However, in *B. germanica* and *P. americana*, TOR and ILP signaling enhanced the expression of *JH methyltransferase* (*JHAMT*), *Met*, and *Kr‐h1*, further stimulating Vg expression and oocyte maturation (Abrisqueta et al., 2014; S. Zhu et al., 2020). In our study, knockdown of *LeMet* and *LeKr‐h1* genes significantly reduced *FoxO* mRNA expression but did not influence *InR* transcript levels. Similar results were also found in *C. suppressalis* (Tang et al., 2020). It was probably because *LeMet* and *LeKr‐h1* did not contribute directly to *InR*. *LeMet* or *LeKr‐h1* may interact with *FoxO* through certain indeterminate mediators to collaborate in regulating vitellogenesis in *L. entomophila*. Further study will be needed to investigate the role of the ILP/TOR pathway in the regulation of vitellogenesis in *L. entomophila*.

In conclusion, two related genes *LeMet* and *LeKr‐h1* were identified from *L. entomophila*. JHIII topical treatment induced the expression of JH signaling and *Vg* genes. Combined with the results of RNAi experiments, it was shown that JH regulated the expression of *Kr‐h1* through *Met* and promoted female ovary development. Our results revealed an important role of *Kr‐h1* in insect ovarian development and egg production. These two genes have the potential for application against this rampant pest based on RNAi or exogenous hormone analogs. Meanwhile, more in‐depth studies of the role of JH and crosstalk with insulin signaling in insect reproduction regulation is well needed.

## AUTHOR CONTRIBUTIONS

**Bin‐Bin Yang**: Conceptualization (equal); data curation (equal); formal analysis (equal); investigation (equal); methodology (equal); writing – original draft (equal); Writing – review and editing (equal). **Shi‐Yuan Miao**: Conceptualization (equal); data curation (equal); formal analysis (equal); methodology (equal); project administration (lead); writing – original draft (equal); writing – review and editing (lead). **Yu‐Jie Lu**: Conceptualization (lead); funding acquisition (lead); project administration (lead); Resources (lead); supervision (lead); writing – review and editing (equal). **Sui‐Sui Wang**: Conceptualization (supporting); data curation (supporting); formal analysis (supporting); methodology (lead); project administration (supporting). **Zheng‐Yan Wang**: Project administration (supporting); resources (supporting); supervision (supporting); writing – review and editing (supporting). **Ya‐Ru Zhao**: Methodology (equal); project administration (equal); writing – review and editing (supporting).

## CONFLICT OF INTEREST

The authors declare no conflict of interest.

## Data Availability

The data that support the findings of this study are available from the corresponding author upon reasonable request.
